# Glucose and cholesterol induce abnormal cell divisions via DAF-12 and MPK-1 in *C. elegans*

**DOI:** 10.18632/aging.103647

**Published:** 2020-08-28

**Authors:** Zhi Qu, Shaoping Ji, Shanqing Zheng

**Affiliations:** 1 School of Nursing and Health, Henan University, Kaifeng 475004, Henan Province, China; 2 School of Basic Medical Sciences, Henan University, Kaifeng 475004, Henan Province, China; 3 Medical School, Henan University, Kaifeng 475004, Henan Province, China

**Keywords:** *C. elegans*, glucose, cholesterol, neuronal cell divisions, MPK-1

## Abstract

People exposed to starvation have a high risk of developing cancer later in life, and prior studies have shown these individuals have high insulin and cholesterol levels and are sensitive to glucose. Using *C. elegans* as a model, we found that glucose and cholesterol can promote survival and cause starved L1 diapause worms to undergo abnormal neuronal cell divisions. Starvation has also been shown to promote long-term survival; however, we found that the functions of glucose and cholesterol in relation to these cell divisions are distinct from their effects on survival. We demonstrate that glucose functions in a DAF-16/FOXO-independent IIS pathway to activate the MAPK ontogenetic signaling to induce neuronal Q-cell divisions, and cholesterol works through DAF-12/steroidogenic pathways to promote these cell divisions. *daf-12* and *mpk-1/MAPK* mutants suppress the function of glucose and cholesterol in these divisions, and a fully functioning dpMPK-1 requires the steroid hormone receptor DAF-12 for these divisions to occur. These afflictions also can be passed on to the immediate progeny. This work indicates a possible link between glucose and cholesterol in starved animals and an increased risk of cancer.

## INTRODUCTION

Starvation induces phenotypic consequences for exposed individuals. Women who experience starvation during pregnancy present a significantly increased risk of breast cancer [1], and offspring born to these mothers also have a higher risk of developing cancer later in life [2, 3]. People exposed to starvation have higher insulin concentrations, different plasma lipid profiles, and reduced plasma protein concentrations compared to unexposed individuals [2, 4–6]. The combination of high insulin and glucose levels is critical to the development of type 2 diabetes; however, why starved animals have high insulin levels and how exactly glucose and other hormones impact tumor cell proliferation in starved animals still requires further investigation. Notably, one recent study has shown that glucose supplementation can promote the survival and stress resistance of starved larval-stage worms [7]. However, hormones and lipids may use different mechanisms to influence diseases, stress resistance, and survival, respectively, in both starvation-exposed animals and their immediate offspring.

The nematode *Caenorhabditis elegans (C. elegans)* has been widely used as a model organism to study the impact of starvation on signal transduction, genetic circuits, and developmental rates [7–11]. In the laboratory, *C. elegans* eggs can be collected and cultured in a sterile environment to induce larval-stage arrest (L1 arrest), which is a diapause that occurs in response to starvation, while genetic regulatory mechanisms allow these worms to recover from this diapause and progress through postembryonic development in the absence of food [12]. As such, *C. elegans* provides an ideal model to study the effects of starvation during genetically normal developmental processes.

In this study, we present evidence that high concentrations of glucose, and steroid hormones (induced by high levels of cholesterol) can result in neuronal Q-cell divisions in L1-arrested *C. elegans* larva. It has been shown that exposure to starvation at an early developmental stage of an animal may affect the formation of the neuronal system, and such changes to neuronal function caused by starvation can increase the animal’s susceptibility to diseases later in life [2]. The mechanisms underlying the effects of glucose and steroid hormones in regards to neuronal cell division have rarely been reported. The insulin/IGF-1 signal pathway (IIS) is negatively regulated by the human tumor suppressor homolog of PTEN (DAF-18) in *C. elegans* [13, 14], loss of *daf-18/PTEN* activates IIS signal transduction in worms, and the IIS pathway is reported to regulate cell divisions in both DAF-16–dependent and –independent manners [8, 15–17]. Our previous study showed that loss of function in *daf-18/PTEN* or overexpression of the insulin agonist (such as *ins-6*) in worms causes neuronal Q-cell divisions during starvation [17, 18]. The IIS pathway and its final transcription factor, DAF-16, primarily function to regulate the survival phenotype induced by glucose supplementation [7, 9, 17]. However, we found that DAF-16 was not responsible for the neuronal Q-cell divisions observed in the glucose and steroid-treated L1-arrested worms. The mechanisms by which glucose and steroid affect cell growth and survival in starved animals appear to be independent. We found that neuronal Q-cell divisions induced by glucose and hormonal steroids can be suppressed by mutations in the nuclear hormone receptor DAF-12. Furthermore, we found that the function of MPK-1, a conserved protein in the RAF-MAPK pathway, can be evoked by glucose and steroid treatments. This study shows that glucose and steroids can induce phosphorylation of MPK-1, which is needed for tumorigenesis, and the activated form of MPK-1 requires DAF-12. This work highlights the pathophysiology of a possible linkage between glucose and steroid hormones and subsequent cancer risk in starved animals.

## RESULTS

### High glucose and cholesterol levels induce neuronal Q-cell divisions during L1 arrest

**Figure 1:**
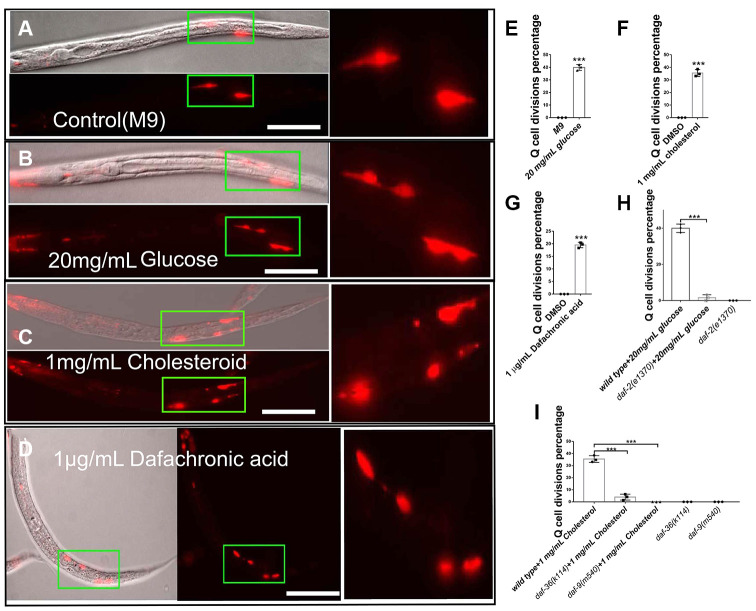
**Glucose, cholesterol, and Δ^7^-dafachronic acid promote neuronal Q-cell divisions in L1-arrested worms.** (**A**) Normal L1-arrested wild-type worms. The wild-type L1-arrested worms only have two Q-cells (QR/L). Wild-type L1-arrested worms treated with 20 mg/mL glucose (**B**, **E**), 1 mg/mL cholesterol (**C**, **F**), or 1 μg/mLΔ^7^-dafachronic acid (**D**, **G**). Q-cell divisions occurred in these worms, as at least four Q-cells were observed under these conditions. See the dosage treatment results in Supplementary Figure 1, and the Q-cell final descendants test in Supplementary Figure 2. (**H**) Q-cell divisions induced by glucose or insulin treatments can be suppressed by *daf-2* mutants. (**I**) Q-cell divisions induced by cholesterol treatment can be suppressed by *daf-9* and *daf-36* mutants. White bar: 50 μm. Data are the average of three independent experiments. Error bars: Standard Deviation (SD). ***: P<0.001.

L1-arrested *C. elegans* larva pause all development events, including cell divisions, which makes them a perfect model to dissect the physiological or pathological effects of external variables. The two embryonic neuronal Q-cells stop dividing in L1-arrested worms (Figure 1A), but we found that treatment with high glucose caused the neuronal Q-cells to start dividing in L1-arrested worms (Figure 1B, 1E, and dosage treatment results in Supplementary Figure 1). Glucose was reported to regulate IIS pathway [7, 19] and *daf-2*, an IIS receptor gene, mutants could significantly suppressed Q-cell divisions during L1 arrest [17]. We speculated that glucose might serve to alter the function of IIS signaling in L1-arrested worms. Next, we tested if glucose could work through the IIS receptor (DAF-2) to induce Q-cell divisions, and we found that *daf-2* mutants suppressed the Q-cell divisions induced by glucose treatments in L1-arrested worms (Figure 1H). We also observed that treatment with high cholesterol (Figure 1C and 1F) or Δ^7^-dafachronic acid (an nuclear hormone receptor, NHR, ligand) (Figure 1D and 1G) caused the Q-cells to divide. The Q-cell divisions were further confirmed by using another marker *zdIs5* (Supplementary Figure 2). Cholesterol is a steroid precursor and is involved in the synthesis of NHR ligands. Two genes, *daf-36* and *daf-9*, control the conversion of cholesterol to NHR ligands in *C. elegans* [20], and we found that both *daf-9* and *daf-36* mutants suppressed the Q-cell divisions caused by cholesterol treatment (Figure 1I). These results suggested that glucose and cholesterol affected the IIS and steroidogenic pathways to induce abnormal neuronal cell divisions in L1-arrested worms.

### Glucose and cholesterol induce cell divisions independent of their effects on survival extension

**Figure 2:**
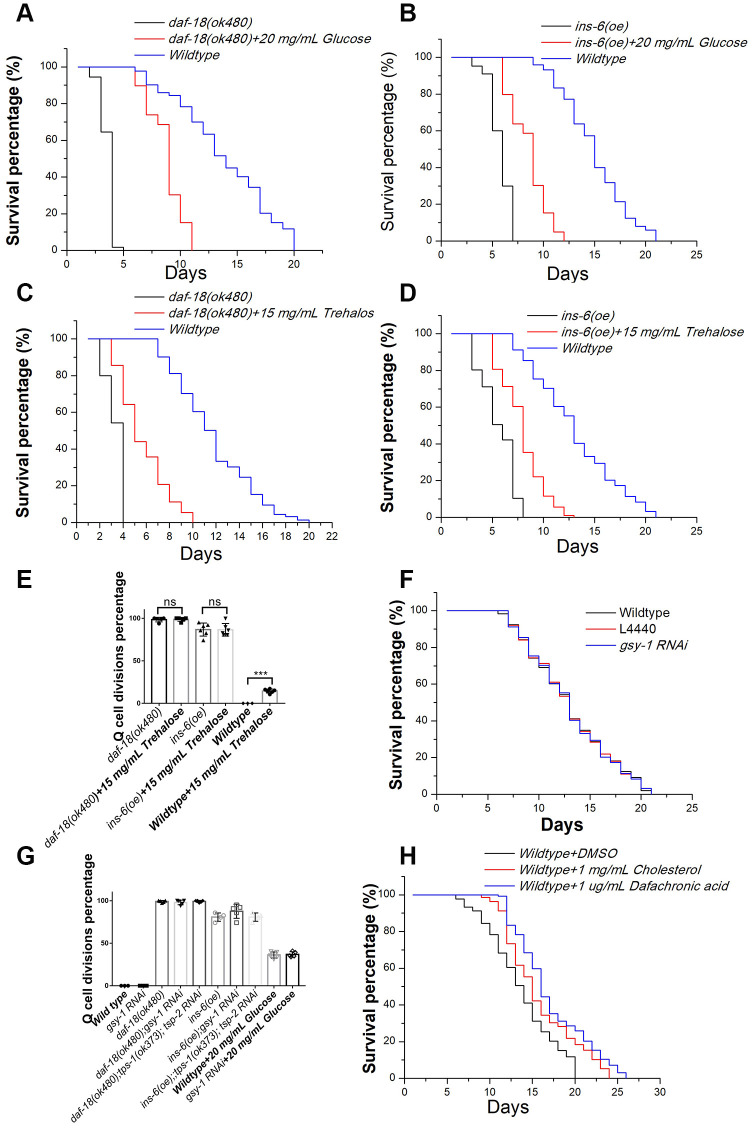
**The effects of glucose, trehalose, cholesterol, and Δ^7^-dafachronic acid on the survival of L1-arrested worms.** Glucose extends the longevity of L1-arrested *ins-6 (oe)* (**A**) and *daf-18* mutants (**B**). Trehalose extends the longevity of L1-arrested *ins-6 (oe)* (**C**) and *daf-18* mutants (**D**). (**E**) Trehalose has no suppression function on Q-cell proliferation; in contrast, it can induce Q-cell proliferation in wild-type L1-arrested worms. Data are the average of at least three independent experiments. Error bars: Standard Deviation (SD).***: P<0.001. (**F**) The glycogen synthesis controlling gene *gsy-1* has no effect on the survival of L1-arrested worms. (**G**) The glycogen and trehalose synthesis controlling genes *gsy-1* and *tps-1/2* have no effect on the Q-cell divisions in *daf-18 (-)*, *ins-6 (oe)*, and wild-type L1-arrested worms. (**H**) Cholesterol and dafchronic acid can extend the survival of wild-type L1-arrested worms. Survival of these worms was checked every day, and the mean survival rate was calculated using the Kaplan-Meier method, and any significant difference in overall survival rates was determined using the log-rank test (P-value; see Supplementary Table 1).

Our results showed that glucose could induce Q-cell divisions through IIS signal pathway during L1 arrest. Previously, we found that manipulating IIS pathway, such as overexpressing agonist insulin like protein genes (such as *ins-6*) or disruption IIS pathway negative regulator *daf-18/pten*, all can induce Q-cell divisions, and also make these worms have a very short L1 lifespan [17, 18, 21]. We found that glucose could induce neuronal Q-cell divisions in L1-arrested worms; however, a recent study has shown that glucose can also promote the survival of wild-type L1 arrested worms [7]. With this in mind, we tested whether glucose could promote the survival of *ins-6 (oe)* and *daf-18* mutants. We found that the longevity of *ins-6 (oe)* and *daf-18* L1-arrested worms was significantly extended following glucose treatment (Figure 2A and 2B). High glucose levels can lead to increased production of glycogen, and the metabolic shift from glycogen to trehalose can make worms live longer [19]. We tested the role of trehalose on Q-cell divisions and L1 survival and found that trehalose promoted L1 survival in *ins-6 (oe)* and *daf-18* L1 mutants (Figure 2C and 2D). However, trehalose failed to suppress the Q-cell divisions in these worms; moreover, it was observed that treatment with trehalose could induce Q-cell divisions in wild-type L1-arrested worms (Figure 2E). Additionally, *gsy-1* plays a key role in the process of glycogen synthesis under high-glucose conditions; however, we observed that *gsy-1* mutant L1-arrested worms had a normal longevity (Figure 2F) and presented with no aberrant cell divisions. The conversion of external trehalose into glucose and back again to trehalose is necessary to produce the observed physiological effects (i.e., survival extension) [22]. *tps-1/2* are the key genes that control trehalose synthesis, but we found that *tps-1;tps-2* worms had no effect on the glucose-induced cell divisions. *tps-1*; *tps-2* also failed to block the aberrant cell divisions in *ins-6 (oe)* or *daf-18* L1 mutants (Figure 2G). These results suggested that high glucose levels could regulate Q-cell divisions, and this effect might be physiologically distinct from glucose metabolism and its role in survival. As hormonal steroids induced by high levels of cholesterol also promote Q-cell divisions in L1-arrested worms, we also assessed the role of cholesterol in regard to L1 survival. We found that cholesterol and Δ^7^-dafachronic acid could also extend the survival of wild-type L1 worms (Figure 2H). Together, these results suggested that high glucose and hormonal steroids could affect the Q-cell divisions; however, their effects on L1 survival involved distinct mechanisms (All survival data details in Supplementary Table 1).

### DAF-12 and MPK-1 block Q-cell divisions in glucose- and cholesterol-treated L1-arrested worms

**Figure 3:**
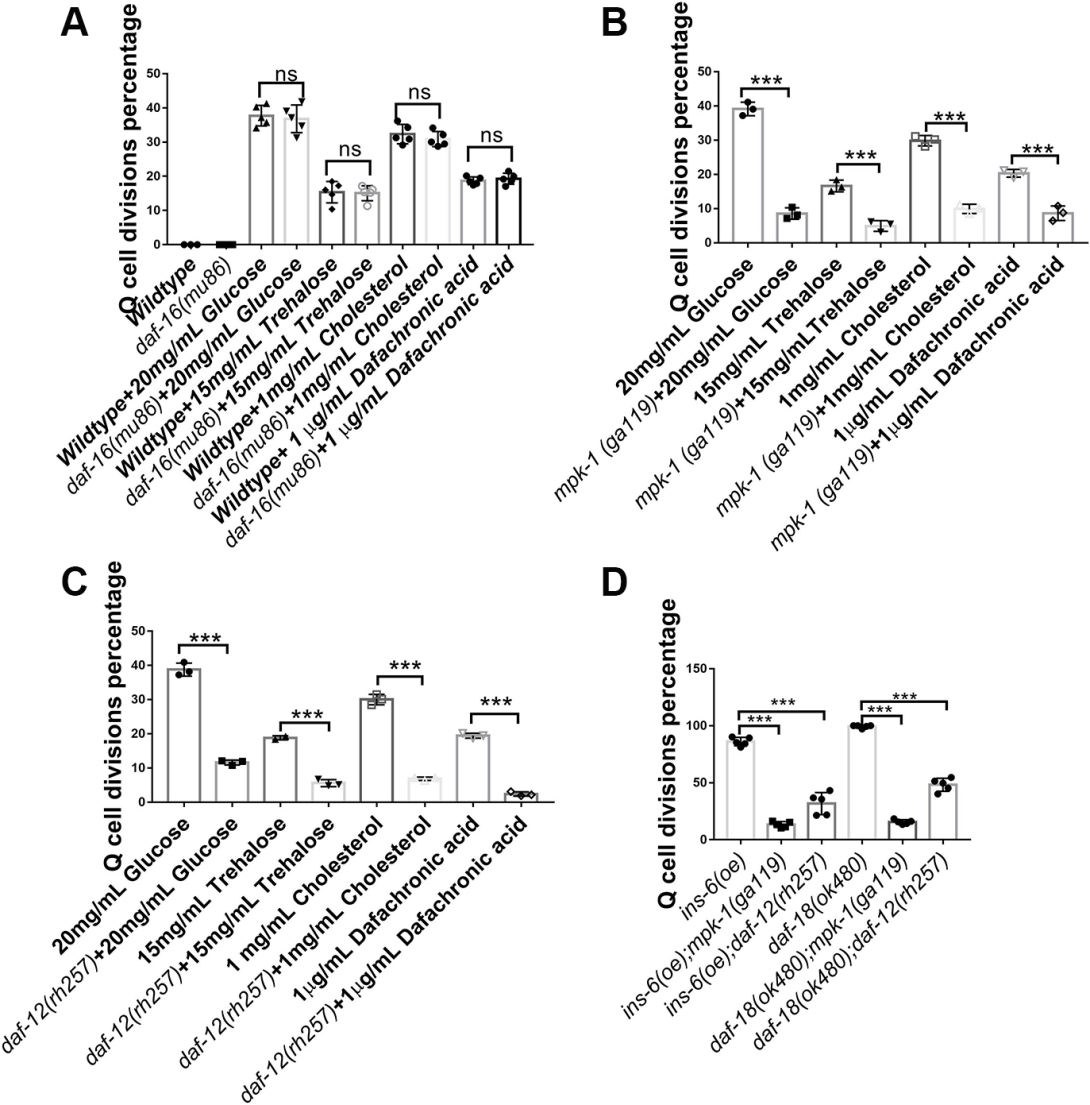
**Neuronal Q-cell divisions in glucose-, trehalose-, cholesterol-, and Δ^7^-dafachronic acid-treated L1-arrested worms are controlled by *daf-12* and *mpk-1*.** (**A**) Q-cell divisions resulting from glucose, trehalose, cholesterol, and Δ^7^-dafachronic acid treatments are not dependent on *daf-16*. Disruption of *mpk-1* (**B**) and *daf-12* (**C**) can suppress the Q-cell divisions in glucose-, trehalose- and steroid-treated wild-type, *ins (oe)*, and *daf-18 (-)* (**D**) L1-arrested worms. More alleles of *daf-12* and *mpk-1* were tested to further confirm these results (Supplementary Figure 3). Data are the average of at least three independent experiments. Error bars: Standard Deviation (SD). ***: P<0.001.

Glucose has been reported to influence a worm’s longevity and stress resistance via the transcription factor DAF-16 of the IIS signaling pathway [22–24]; however, we found that disruption of the *daf-16* gene did not alter the Q-cell divisions occurring in glucose-, trehalose-, or cholesterol-treated worms (Figure 3A). These results suggested that the IIS pathway uses distinct downstream signals (DAF-16 and DAF-16-independent) to regulate survival and cell divisions during L1 arrest. Previously, we observed that *mpk-1/MAPK* played a key role in controlling Q-cell divisions in *daf-18* L1-arrested mutants [17]. Based on this result, we treated *mpk-1* mutants with high concentrations of glucose, trehalose, cholesterol, or Δ^7^-dafachronic acid, and the results indicated that the Q-cell divisions were suppressed in the *mpk-1* mutant worms (Figure 3B). Hormonal steroids and Δ^7^-dafachronic acid mainly function by way of the DAF-12 hormone receptor in *C. elegans*. As such, we tested the role of *daf-12* on Q-cell divisions and found that these cell divisions in glucose-, trehalose-, cholesterol-, or Δ^7^-dafachronic acid-treated worms and *daf-18 (-)* mutants were suppressed by disruption of *daf-12* (Figure 3C). Glucose and trehalose might affect insulin-related gene expression to regulate the IIS pathway and control the Q-cell divisions. As such, we also tested *ins-6 (oe)* and *daf-18* mutants and found that disruption of *mpk-1* or *daf-12* indeed suppressed the Q-cell divisions in these two strains (Figure 3D). In order to further confirm the functions of *daf-12* and *mpk-1*, more alleles of these gene mutations were tested and showed the similar results (Supplementary Figure 3). These results suggested that DAF-12 and MPK-1 work together to regulate the Q-cell divisions induced by glucose and cholesterol in L1-arrested worms.

### dpMPK-1 plays a major role in neuronal Q-cell divisions induced by glucose and cholesterol treatments during L1 arrest

**Figure 4:**
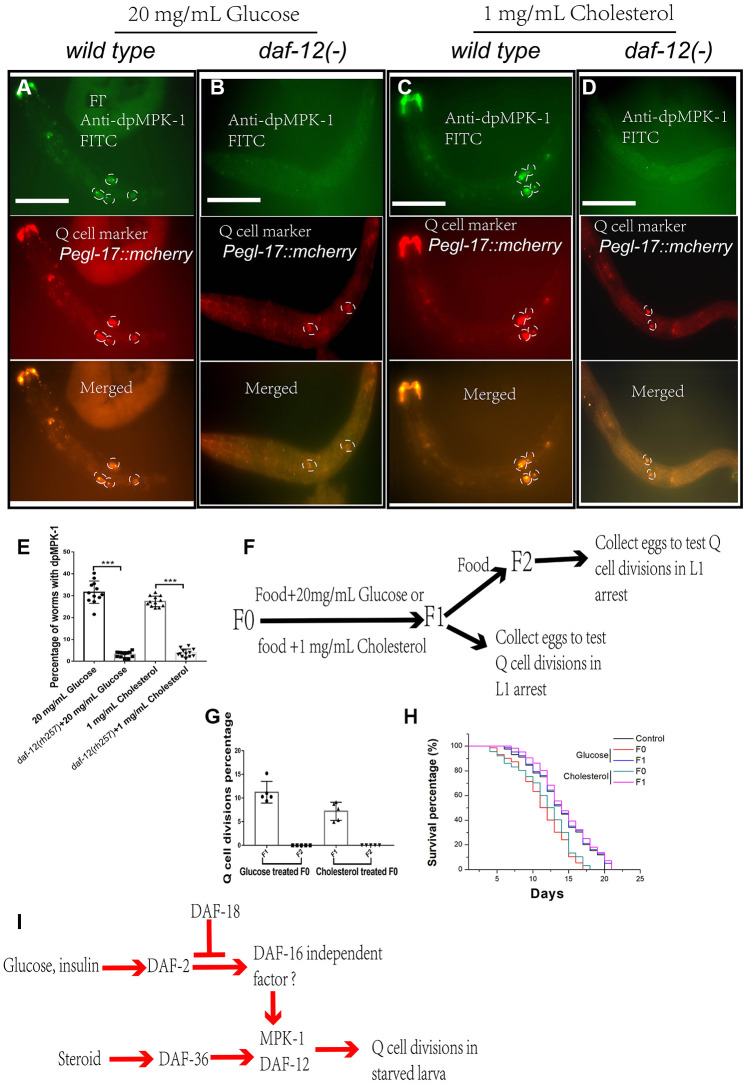
**dpMPK-1 is activated by glucose and cholesterol, and *daf-12* is needed to induce Q-cell divisions.** The activated form of MPK-1 (dpMPK-1) is detected in glucose- (**A**) and cholesterol- (**C**) treated L1-arrested worms. *daf-12(-)* can suppress Q-cell divisions and block dpMPK-1 formation in glucose- (**B**) and cholesterol- (**D**) treated L1-arrested worms. (**E**) Worms with and without dpMPK-1 fluorescence were counted, and the percentage of worms expressing dpMPK-1 was calculated. Data are the average of at least ten independent experiments (sample size >50 in each experiment). Error bars: Standard Deviation (SD). ***: P<0.001. White bar: 50 μm. (**F**) The glucose and cholesterol treatment is only performed in F0-F1 culture period. F1 and F2 worms were collected and assessed for Q-cell divisions. (**G**) The F1 progeny of glucose- and cholesterol-treated worms also present aberrant Q-cell divisions, but the F2 generation shows no abnormal Q-cell division. Data are the average of at least three independent experiments (sample size >50 in each experiment). Error bars: Standard Deviation (SD). (**H**) The survival of the F1 and F2 progeny of F0 worms treated with glucose and cholesterol. (**I**) Working model: cholesterol and glucose can work through DAF-12 and DAF-16 independent of the IIS pathway to activate MPK-1 to induce neuronal Q-cell divisions in L1-arrested worms, and these effects can also affect the F1 generation.

MPK-1 is reported to be the main regulator controlling Q-cell proliferation during L1 arrest [17], and dpMPK-1 is the functional form of MPK-1 that acts during cell proliferation [17, 25–27]. With this in mind, we tested whether high glucose or cholesterol treatments could affect dpMPK-1 levels. We found that glucose- and cholesterol-treated L1-arrested worms both had high dpMPK-1 levels during cell divisions (Figure 4A, 4C, and 4E). DAF-12 and MPK-1 can both suppress the Q-cell divisions during L1 arrest. Moreover, DAF-12 is a nuclear hormone receptor, and MPK-1 can translocate into the nucleus. As such, we assess dpMPK-1 levels in *daf-12* mutants and found that the dpMPK-1 levels, which were up-regulated by glucose and cholesterol, were rescued by mutation of *daf-12* (Figure 4B, 4D, and 4E). These results suggest that the function of dpMPK-1 on Q-cell divisions requires the nuclear hormone receptor DAF-12.

Mothers who experience starvation have high blood insulin concentrations and are also reported to have babies with poor health who have a high chance of developing diseases, including cancer [2, 3, 28]. We wanted to determine if high glucose or steroid levels could influence subsequent generations of worms. To this end, we tested whether the decedents of high glucose- or cholesterol-treated worms still underwent Q-cell divisions during L1 arrest. We found the F1 L1-arrested worms also presented with aberrant Q-cell divisions (Figure 4F and 4G) and a shortened survival time (Figure 4H), but the F2 generation of worms was essentially wild type (Figure 4G and 4H). These results suggested that the effects of high glucose or steroid treatments in regard to these cell divisions could be passed on to the F1 generation, though this was absent in the F2 generation.

## DISCUSSION

Diet and metabolic genes can modify the levels of hormones in animals and subsequently change their physiology, and recent studies have shown that dietary restriction and food deprivation can promote longevity, stress resistance, and anti-inflammation [29–31]. However, this starvation stratagem should be carefully considered on an individual basis.

In this study, we demonstrated that high glucose might activate MAPK tumorigenic signaling in neurons (Figure 4I). MAPK is activated by starvation in the pharynx muscles of worms [32], and the pharyngeal muscle physiology might subsequently be altered by MAPK to adjust for dietary and starvation conditions. Such alterations to the diet might be passed to the progeny of these animals, as the progeny of glucose-treated mother worms display abnormal Q-cell divisions. Rapid postnatal growth following fetal growth restriction has been associated with permanently raised levels of growth factors, lipid molecules, and other hormones, including steroids. Importantly, steroids play critical roles in a number of disorders and cancers [33–37]. Steroid biosynthesis is an anabolic pathway that produces steroids from simple precursors, making this pathway a common target for antibiotics and other anti-infection drugs [38]. In response to differences in nutrient availability from different diets, metabolic networks are modulated to meet cellular and organismal needs.

Wild-type worms present with an extended lifespan if their great-grandparents were exposed to starvation-induced developmental arrest [11, 39]. Additionally, glucose- and trehalose-treated L1-arrested worms also show an extended lifespan [7]. However, the survival promotion induced by glucose and trehalose is independent of their effect on cell divisions [7]. The IIS pathway is connected to starvation-induced survival and cell proliferation [8, 9, 17, 18, 21, 22], and the transcription factor DAF-16 in this pathway has been shown to play a role in controlling glucose- and stress-induced longevity [7]. However, the neuronal Q-cell proliferation phenotype is not dependent on DAF-16, as shown in this and previous studies [8, 17]. The levels of the ceramide in L1-arrested worms have also been reported to regulate stress resistance in both IIS-dependent and -independent manners [6], as ceramide secretion can induce many pathogen-inducible genes in L1-arrested worms. The mechanisms that control survival, cell proliferation, and other metabolic diseases might be different from each other, and whether glucose or steroids have any connection with ceramide in the regulation of cell divisions and survival still requires further investigation.

This study showed that glucose and steroids (i.e., cholesterol), can induce an oncogenic-like response in animals exposed to starvation and the first generation of their progeny (Figure 4I). The starvation-induced metabolism and cell-division responses in the current generation and immediate offspring could be of benefit to subsequent generations, as starvation early in life can improve the growth rate, fertility, and stress and starvation resistance of the subsequent progeny over multiple generations [10, 16, 40]. Protein-coding genes and mRNA isoforms undergo dramatic changes in starved worms, and alternative isoforms of mRNAs and their expression can have post-transcriptional consequences [41, 42]. Additionally, starvation alters chromatin modification, resulting in transgenerational epigenetic modifications that serve to protect the progeny [10]. Studies on starvation using diverse animal species have provided evidence that epigenetic modification of the genome in malnourished parents can be passed on to the immediate offspring [28, 43, 44], and a variety of life-history traits, including survival promotion and stress resistance, can be epigenetically inherited over multiple generations [10, 11, 40, 45]. Whether these modifications can influence cancer and other metabolic diseases and how these modifications, including changes in RNA generation, metabolism, signal transduction, and epigenetic genome modifications, cooperate with sugar, lipids, and hormone levels to regulate survival and cell proliferation in the immediate and subsequent generations still requires further investigation.

## MATERIALS AND METHODS

### Strains

The strains used in this study were acquired from the Caenorhabditis Genetics Center (CGC) and were crossed into P*egl-17*-mCherry (*rdvIs1*) to assess Q-cell divisions. Standard culture methods were used as previously described [46]. Strains were maintained on worm NGM plates and cultured with OP50 *Escherichia coli* at 20°C. The strains used in this study were RDV55: *rdvIs1*, RB712: *daf-18*(ok480), SD420: *mpk-1*(ga119)/*dpy-17*(e164)*unc-79*(e1068), VC255: *tps-1*(ok373), AA1: *daf-12*(rh257), CB1370: *daf-2* (e1370), DR2281: *daf-9* (m540), and AA292: *daf-36* (k114).

### Chemicals and treatments

Glucose, trehalose, Δ^7^-dafachronic acid and cholesterol were bought from Sigma-Aldrich (USA). Glucose and trehalose were dissolved in water, while the other compounds were dissolved in DMSO. Serial dilutions of each chemical were added into M9 right after the embryos were prepared, so the worms were hatched in a drug-treatment environment and influenced by the chemical prior to L1 arrest.

### Q-cell division analyses

Normally fed, mixed-staged worms were harvested and bleached to prepare the embryos, as previously described [17]. In brief, embryos were maintained and hatched in sterile M9 and incubated at 20°C with low-speed rocking to initiate L1 arrest. Q-cell descendants were observed under an Axioplan fluorescent microscope (Zeiss) after 12 hours in L1 arrest. A total of 50-200 μL M9 containing L1-arrested worms were removed from the culture each time to make sure the sample was larger than 50 worms. The total number of worms and the worms presenting with Q-cell divisions were counted. Q-cell divisions were confirmed via lineage analysis using the Q-cell marker P*egl-17*::mCherry (*rdvIs1*). For transgenic strains, only the worms with the injection marker (*odr-1::gfp*) were counted and analyzed.

### L1 longevity analysis

Life span was assessed in liquid medium. L1 worms were cultured in 1 mL M9, and 50-100 μL was taken to ensure the sample size was larger than 50 worms. The worms were scored every day by counting the number of worms that were moving (alive) and then dividing that number by the total number of worms in the aliquot. To compare the survival rates between strains, the assay was carried out in triplicate with at least 100 L1s, and the mean survival rate was calculated using the Kaplan-Meier method [21] to determine the fraction of living animals over the time course of the experiment. Any significant difference in the overall survival rates was determined using the log-rank test.

### Transgenic strains

For the INS-6 overexpression strains, the gene sequence was amplified from *C. elegans* genomic DNA and placed under the control of the pan-neuronal promoter P*rgef-1*. A plasmid with the injection marker *odr-1::rfp* was injected into P*egl-17*::mCherry (*rdvIs1*) worms using standard microinjection methods (Mello et al., 1991), and at least three stable lines were selected for each injected strain.

Primer sequences used for *ins-6* amplification: Forward 5’-aattgctagcATGAACTCTGTCTTTACTATCATCTTCG-3’, Reverse 5’-aattggtaccTCATGGACAACAAGCAGATCTTATG-3’.

### Antibody staining

Antibody staining was performed as previously described [47]. In brief, L1 worms were collected in 100 μl M9, fixed with 200 μl of cold 2X witches brew and 100 μl 10% paraformaldehyde, and then incubated at 4°C for 30 min to overnight. The worms were washed twice in Tris-Triton buffer, incubated in 1% ßME/Tris-Triton for 1-2 hours at 37 °C, and then washed in 1X Borate buffer. Subsequently, the worms were incubated in 10 mM DTT/1X Borate buffer for 15 min at room temperature, washed in 1X Borate buffer, incubated in 0.3% H_2_O_2_/1X Borate buffer for 15 min, incubated for 15 min in PBST-B, and then washed with PBST-A. The worms were then visualized using an Axioplan fluorescent microscope (Zeiss, Germany).

For dpMPK-1 observation and antibody staining, the L1-arrested worms were collected at 10, 20, 40, and 48 hours after the embryos were prepared. We found that 40 hours after the embryos were placed in M9 buffer was the best time to detect dpMPK-1. Worms with more than two cells detected using anti-dpMPK-1 were scored. The anti-dpMPK-1 [26] and all secondary antibodies were purchased from Sigma-Aldrich (USA).

### RNAi

An RNAi bacterial strain (HT115) expressing a double-stranded target RNA (vector, L4440) was cultured and used to inactivate the target gene function. Eggs from RNAi fed worms were transferred to fresh NGM plates containing the same bacteria and allowed to grow at 15°C for 3 days. Stage 4 larva were then transferred to the same RNAi NGM plates and cultured at 20°C for 24 hours. The RNAi NGM plates contained 1 mM isopropyl-B-D-thiogalactopyranoside (IPTG) for the induction of the double-stranded RNA. The L4440 carrying the empty RNAi plasmad was used as a negative control in RNAi experiments.

## Supplementary Material

Supplementary Figures

Supplementary Table 1
