# Supplementary material for: Glucose and cholesterol induce abnormal cell divisions via DAF-12 and MPK-1 in C. elegans
Source: Aging (Albany NY). 2020 Aug 28;12(16):16255–69. doi: 10.18632/aging.103647 (PMC7485695; doi:10.18632/aging.103647)
Supplement: Supplementary Figures [file aging-12-103647-s002..pdf]

SUPPLEMENTARY FIGURES

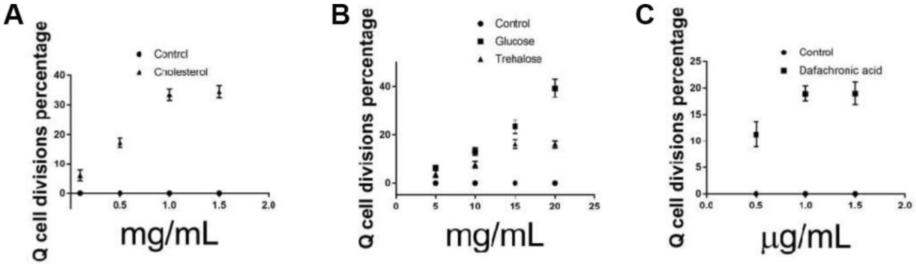

Supplementary Figure 1. Q-cell divisions in worms treated with different concentrations of cholesterol (**A**), glucose (**B**), trehalose (**B**) and dafachronic acid (**C**). Data are the average of three independent experiments. Error bars: Standard Deviation (SD).

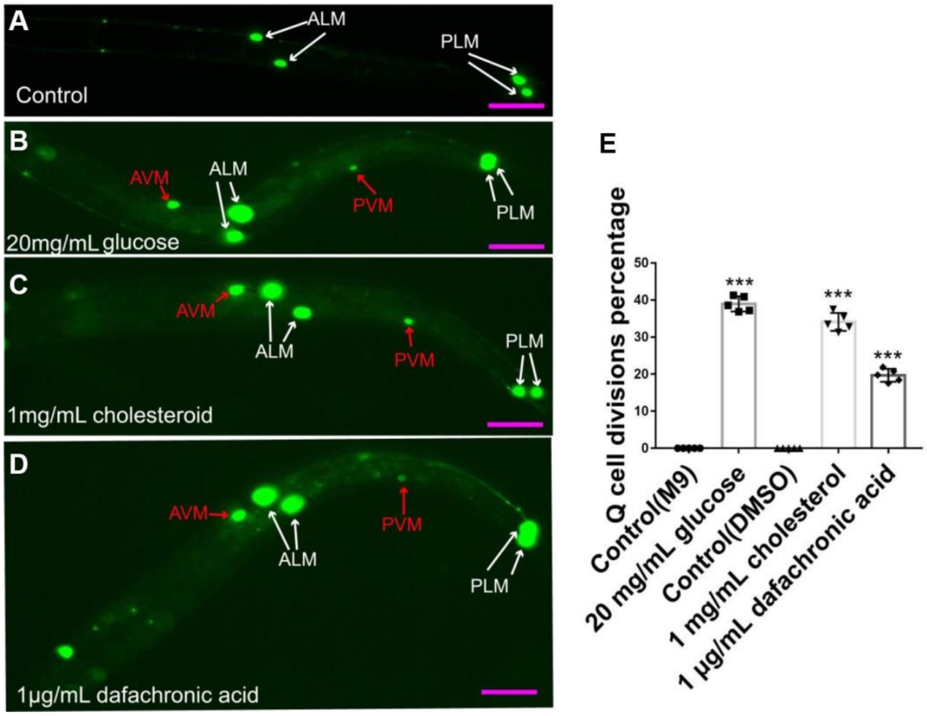

Supplementary Figure 2. (**A**) Q-cell divisions induced by glucose (**B**), cholesterol (**C**) and dafachronic acid (**D**) were further confirmed by using another marker *zdIs5*. Q-cells have 6 final descendants: AVM, PVM, A/PQR and SDQR/L. Only AVM and PVM are touch neurons. *zdIs5* is a touch neuron marker, can light up all 6 touch neurons including AVM, PVM, 2 ALMs and 2 PLMs. ALMs and PLMs are embryonic cells, so they already born before L1 developmental stage (**A**). Data are the average of five independent experiments (**E**), the total number of sample in one test is larger than 150. Error bars: Standard Deviation (SD). Pink bar: 50 μm. ***: P<0.001.

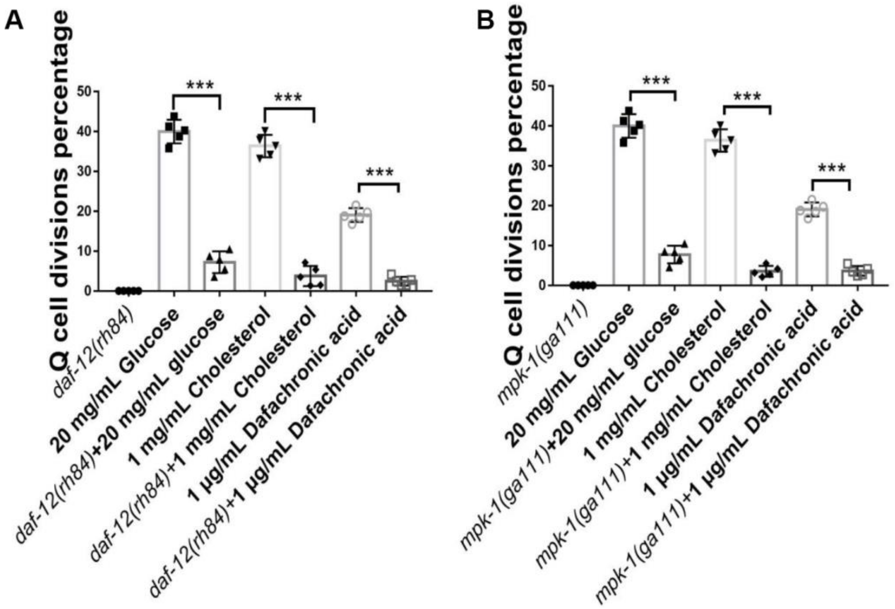

Supplemenatry Figure 3. Q-cell divisions induced by glucose and cholesterol are suppressed by *daf-12* and *mpk-1* mutations. In order to further confirm our conclusion, we used more alleles to test the functions of *daf-12* and *mpk-1* on Q-cell divisions induced by glucose, cholesterol and dafachronic acid. New alleles of *daf-12 (rh84)* (**A**) and *mpk-1 (ga111)* (**B**) were used. Our results further confirmed our conclusion. Data are the average of five independent experiments. Error bars: Standard Deviation (SD). ***: P<0.001.

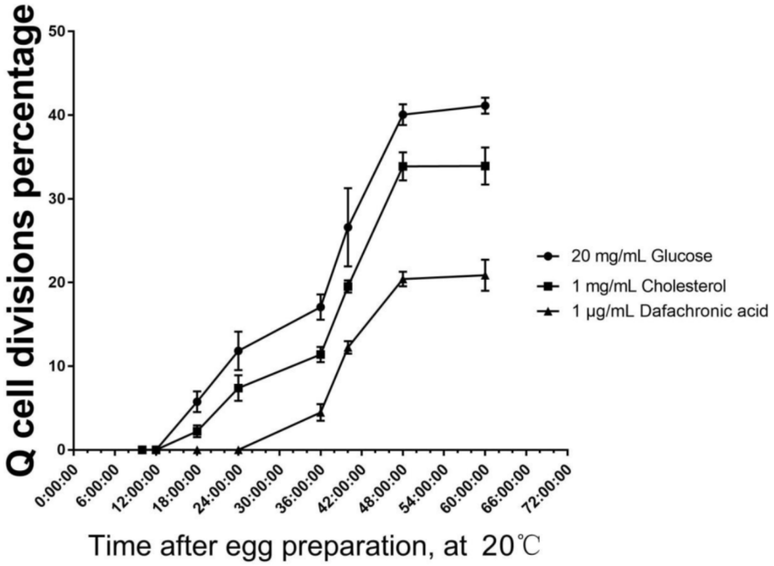

Supplementary Figure 4. Elapsed times of Q-cell divisions in L1 arrest worms treated with glucose, cholesterol and dafachronic acid. The percentages of Q-cell divisions at different time points (10, 12, 18, 24, 36, 40, 48 and 60 hours) after egg preparation. Sample size larger than 100, three independent experiments were analyzed at each time point, error bars show standard divisions.
