# Supplementary material for: Glucose and cholesterol induce abnormal cell divisions via DAF-12 and MPK-1 in C. elegans
Source: Aging (Albany NY). 2020 Aug 28;12(16):16255–69. doi: 10.18632/aging.103647 (PMC7485695; doi:10.18632/aging.103647)
Supplement: Supplementary Table 1 [file aging-12-103647-s001..pdf]

SUPPLEMENTARY TABLE

Supplementary Table 1. Survival data.

	mean±SE	P vs wildtype	P vs *daf-18*
**wildtype**	13.68±0.87		
daf-18	3.01±1.06	<0.001	
***daf-18* +Glucose**	8.75±0.23	<0.001	<0.001
	**mean±SE**	**P vs wildtype**	**P vs daf-18**
**wildtype**	14.25±0.41		
daf-18	3.01±1.06	<0.001	
***daf-18* +Trehalose**	7.13±1.09	<0.001	<0.001
	**mean±SE**	**P vs wildtype**	**P vs ins-6 oe**
**wildtype**	13.98±0.25		
ins-6 oe	5.01±0.16	<0.001	
***ins-6 oe* +Glucose**	9.38±1.08	<0.001	<0.001
	**mean±SE**	**P vs wildtype**	**P vs ins-6 oe**
**wildtype**	13.79±1.03		
ins-6 oe	5.01±0.16	<0.001	
***ins-6 oe* +Trehalose**	8.05±0.68	<0.001	<0.001
	**mean±SE**	**P vs wildtype**	
**wildtype**	14.02±0.65		
**Cholesterol**	17.0±0.45	<0.001	
**Dafachronic acid**	15.84±1.01	<0.001	
	**mean±SE**	**P vs wildtype**	
**wildtype**	14.56±0.89		
**F0**	11.35±0.54	<0.01	
**F0**	12.97±0.14	<0.01	
**F1**	14.67±1.05	#	
**F1**	14.35±0.15	#	
	**mean±SE**	**P vs wildtype**	
**wildtype**	14.01±0.25		
gsy-1 RNAi	13.78±1.11	#	

Survival of these worms was checked every day, and the mean survival rate was calculated using the Kaplan-Meier method, and any significant difference in overall survival rates was determined using the log-rank test (P value). #: no significant difference. Glucose: 20 mg/mL, Cholesterol: 1 mg/mL, Δ7-dafachronic acid: 1 μg/ mL, Trehalose: 15 mg/mL.
